# Antibacterial performance of Ag nanoparticles and AgGO nanocomposites prepared via rapid microwave-assisted synthesis method

**DOI:** 10.1186/1556-276X-7-541

**Published:** 2012-09-28

**Authors:** Soon Wei Chook, Chin Hua Chia, Sarani Zakaria, Mohd Khan Ayob, Kah Leong Chee, Nay Ming Huang, Hui Min Neoh, Hong Ngee Lim, Rahman Jamal, RahaMohdFadhilRajaAbdul Rahman

**Affiliations:** 1 School of Applied Physics, Faculty of Science and Technology, Universiti Kebangsaan Malaysia, Bangi, Selangor, 43600, Malaysia; 2 School of Chemical Sciences and Food Technology, Faculty of Science and Technology, Universiti Kebangsaan Malaysia, Bangi, Selangor, 43600, Malaysia; 3 Physics Department, Faculty of Science, University of Malaya, Kuala, Lumpur, 50603, Malaysia; 4 UKM Medical Molecular Biology Institute, Universiti Kebangsaan Malaysia, Cheras, Kuala Lumpur, 56000, Malaysia; 5 Department of Chemistry, Faculty of Science, Universiti Putra Malaysia, UPM Serdang, Selangor, 43400, Malaysia

**Keywords:** Antibacterial properties, Graphene oxide, Microwave irradiation, Nanocomposites, Silver nanoparticles

## Abstract

Silver nanoparticles and silver-graphene oxide nanocomposites were fabricated using a rapid and green microwave irradiation synthesis method. Silver nanoparticles with narrow size distribution were formed under microwave irradiation for both samples. The silver nanoparticles were distributed randomly on the surface of graphene oxide. The Fourier transform infrared and thermogravimetry analysis results showed that the graphene oxide for the AgNP-graphene oxide (AgGO) sample was partially reduced during the *in situ* synthesis of silver nanoparticles. Both silver nanoparticles and AgGO nanocomposites exhibited stronger antibacterial properties against Gram-negative bacteria (*Salmonella typhi* and *Escherichia coli*) than against Gram-positive bacteria (*Staphyloccocus aureus* and *Staphyloccocus epidermidis*). The AgGO nanocomposites consisting of approximately 40 wt.% silver can achieve antibacterial performance comparable to that of neat silver nanoparticles.

## Background

Silver (Ag) is well known as an effective antibacterial material for treating wounds and chronic diseases [1]. It exhibits strong cytotoxicity against a broad range of microorganisms; however, the conventional usage of silver salt and silver metal, which may release the silver ion too rapidly or too inefficiently in silver releasing, has limited its biomedical applications [2]. Therefore, silver nanoparticles (AgNPs) that possess high specific surface area and unique physicochemical properties have attracted abundance of interest in various fields [3]. Nowadays, AgNP has been widely used for coating of medical devices, wound dressing, water filtration, etc. [1,4].

It has been reported that chemical reduction is one of the most popular methods for the preparation of AgNP due to its simplicity, low cost, and ability to produce a large amount of sample [5]. In this process, a reducing agent is needed to initiate the formation of AgNP. Various types of green reducing agents have been studied for the synthesis of AgNP, such as chitosan [6] and sugars [7], plant extracts [8], and bacterium [9]. One of the simplest green synthesis methods was based on Tollens' process and uses glucose to form stable colloidal AgNP [10].

A microwave-assisted method had been reported for the synthesis of AgNP [11,12] since it is well known as a rapid process in producing metallic nanoparticles, for example, gold, platinum, and palladium [13]. The microwave chemistry involves a dipolar mechanism and ionic conduction [14,15]. Monodispersed nanoparticles with high crystallinity and small and uniform size distribution can be produced using microwave irradiation due to the homogeneous heating of the reaction medium, which improves the reaction rate to hasten the fast nucleation and crystal growth of nanoparticles [11,15]. In addition, microwave-assisted synthesis only requires lower energy consumption compared to conventional heating method [11,16].

Recent studies suggested that graphene oxide (GO) possesses antibacterial properties against *Escherichia coli*[17-19] and that AgNP-functionalized graphene-based materials exhibit enhanced antibacterial properties [20-23]. GO is a sheet of *sp*^2^-bonded single-carbon-atom-thick graphene which was chemically functionalized with oxygen functional groups such as carboxylic and carbonyl at the edges of the sheet, and epoxy and hydroxyl on the basal plane [24-27]. With the presence of the oxygen functional groups, exfoliated GO is well dispersed in polar solvents, such as water [28]. This hydrophilic property allows the deposition of metallic nanoparticles and, subsequently, the utilization in various applications [22,25,29-33].

In the present study, AgNP and AgNP-graphene oxide (AgGO) nanocomposites were prepared using the microwave approach as a rapid synthesis method by using glucose as a green reducing agent. The antibacterial properties of both samples against Gram-positive and Gram-negative bacteria were investigated. The aim of this study is to produce a new nanocomposite material with lower silver content and comparable antibacterial performance.

## Methods

### Materials

The analytical-grade silver nitrate (AgNO_3_), sodium hydroxide (NaOH), ammonia, potassium permanganate (KMnO_4_, 99.9%), hydrogen peroxide (H_2_O_2_, 30%), sulfuric acid (H_2_SO_4_, 98%), phosphoric acid (H_3_PO_4_, 85%), and glucose used in this study were purchased from Merck (Darmstadt, Germany). Graphite flakes were purchased from Asbury Graphite Mill, Inc. (Asbury, NJ, USA). GO was prepared using the simplified Hummer's method [34]. Briefly, graphite was oxidized to graphite oxide with H_2_SO_4_ and KMnO_4_, and H_2_O_2_ was added to stop the oxidation. Graphite oxide was washed with a simple decantation of the supernatant with repeated centrifugation until pH 7.0 was achieved, and followed by exfoliation in an ultrasonication bath.

### Synthesis of AgNP and AgGO nanocomposites

AgNPs were synthesized using the modified Tollens' process. Briefly, silver oxide was formed by mixing AgNO_3_ (2 mM) and NaOH (2 mM). Then, the silver oxide was dissolved in ammonia solution (10 mM) to form silver ammonia complex. The reaction was followed by reduction using glucose (10 mM) in a commercial microwave oven (NN-SM330M, 700W, Panasonic, Osaka, Japan) for 60 s. After the reaction, the color of the solution turned into greenish-yellow, indicating the formation of AgNP. As to the synthesis of AgGO nanocomposite, silver ammonia complex was mixed with GO suspension (2.5 mg/ml) while stirring for 5 min and followed by the addition of glucose solution (1 mM). The mixture was then put into the microwave oven for 60 s. Both AgNP and AgGO nanocomposites were washed with deionized water by centrifugation to remove excess chemicals.

### Characterizations

The UV-visible (UV-vis) spectrum and X-ray diffraction pattern of the AgNP and AgGO samples were obtained using a UV-vis spectrophotometer (Jenway 7315, Staffordshire, UK) and an X-ray diffractometer (Bruker Advance, Madison, WI, USA) to identify the formation of AgNP. The size and distribution of AgNP for both samples were examined using a scanning transmission electron microscopy (HD-2700 Cs-Corrected FE-STEM, Hitachi, Tokyo, Japan). The silver content of the AgGO sample was estimated using energy dispersive X-ray spectroscopy (EDS) and induced coupled plasma optical emission spectroscopy (Optima 4300 DV, Perkin Elmer, Waltham, MA, USA).

The chemical functional groups of GO and AgGO were characterized using attenuated total reflectance Fourier transform infrared (Perkin Elmer Spectrum 400). The thermal properties of GO and AgGO were measured using a Pyris 1 thermogravimetry analyzer (TGA; Perkin Elmer). The AgGO sample was characterized using X-ray photoelectron spectroscopy (Axis Ultra DLD, Kratos/Shimadzu, Kyoto, Japan).

### Antibacterial test

The antibacterial activity of the AgNP and AgGO were tested on Gram-positive (*Staphyloccocus aureus* and *Staphyloccocus epidermidis*) and Gram-negative (*E. coli* and *Salmonella typhi*) bacteria. The bacteria (10^5^ CFU) were inoculated in nutrient broth and incubated with AgNP and AgGO samples at five different concentrations (6.25 to 100 μg/ml) at a volume ratio of 1:1 for 4 h at 37°C. After the incubation, 0.1 ml of the mixture for each sample was spread on a nutrient agar plate, followed by incubation at 37°C for another 24 h. Control sample (sterilized distilled water) and 100 μg/ml GO were prepared and spread on an agar plate for standard comparison. All the agar plates were visually inspected for the presence of bacterial growth, and the results were recorded.

## Results and discussion

### Formation of AgNP and AgGO nanocomposites

**Figure 1:**
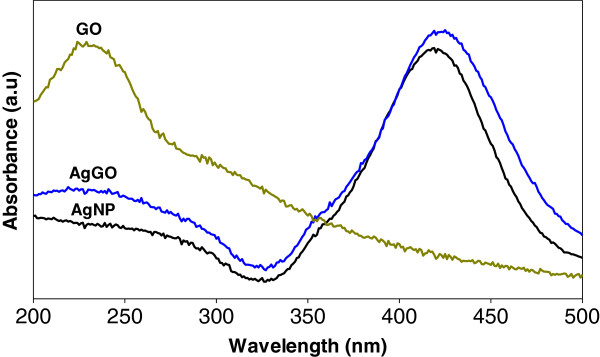
UV-vis spectra of GO, AgNP, and AgGO.

The UV-vis spectra of GO, AgNP, and AgGO are shown in Figure 1. The GO sample shows a strong absorption peak at 230 nm, which is due to the π → π* transitions of aromatic C-C bonds, while n → π* transitions of C=O bonds contribute to the shoulder at 300 nm [35]. Both AgNP and AgGO show a strong absorption peak at 418 and 420 nm, respectively, due to the surface plasmon resonance of AgNP. This phenomenon happened when the incident light interacted with the valence electrons at the outer band of AgNP, leading to the oscillation of electrons along with the frequency of the electromagnetic source [36]. There is a broad absorption range for the AgGO sample at 210 to 240 nm, which can be attributed to the presence of GO.

**Figure 2:**
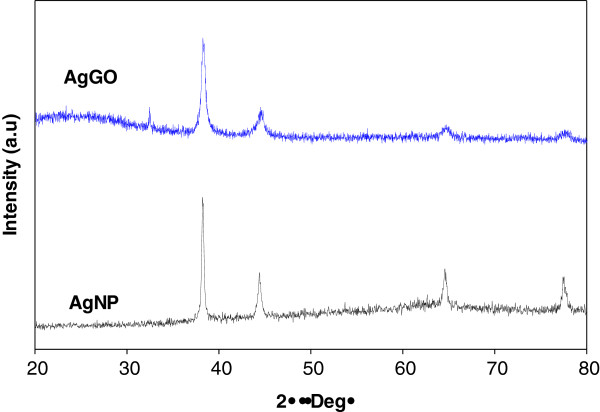
XRD diffraction patterns for AgNP and AgGO.

Figure 2 shows the X-ray diffraction spectra of the AgNP and AgGO samples. The formation of AgNP was confirmed by the existence of diffraction patterns of silver crystal structure, matching with the standard X-ray diffraction (XRD) pattern (JCPDS no. 04-0783). The diffraction peaks for both samples at 38.1°, 44.3°, 64.5°, and 77.4° represent the crystallographic planes of (111), (200), (220), and (311) for the face-centered cubic of the silver crystal. These have further confirmed the formation of Ag crystals in both samples by using microwave irradiation method.

**Figure 3:**
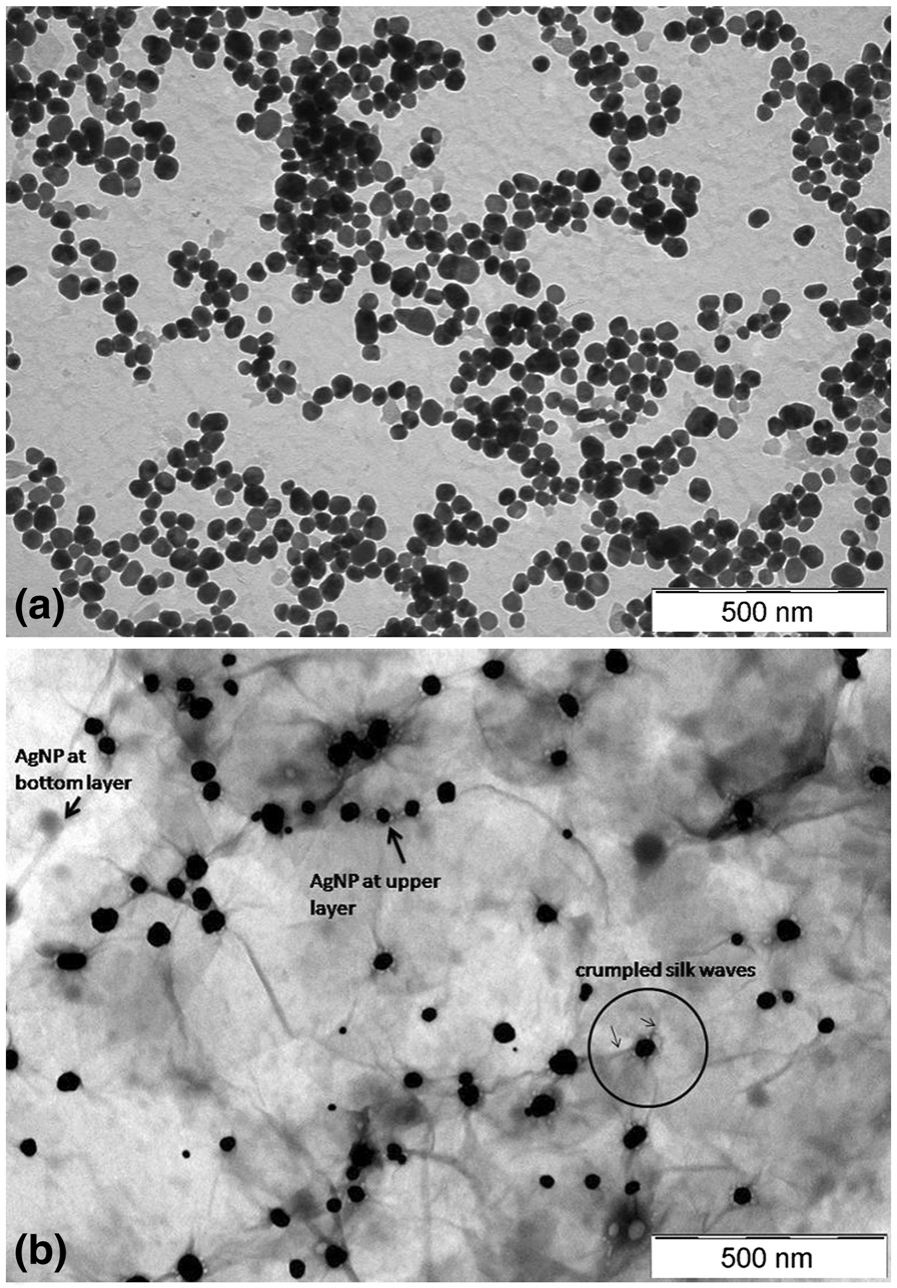
Electron microscopy images of the (a) AgNP and (b) AgGO.

The electron microscopy images of the AgNP and AgGO nanocomposites are shown in Figure 3. Figure 3a reveals the spherical shape of AgNPs with an average size of 37.1 ± 5.1 nm. For the AgGO sample (Figure 3b), it can be seen that the AgNPs were deposited randomly on the GO sheets, and the AgNPs have an average size of 40.7 ± 7.5 nm. The presence of crumpled silk waves on the GO sheets can also be observed, which can be attributed to the deposition of AgNP on the surface of the GO sheets (circle area in Figure 3b). The deposition of AgNP on the upper and bottom layers of the translucent GO sheets can be differentiated by the black-and-white contrast of the particles.

**Figure 4:**
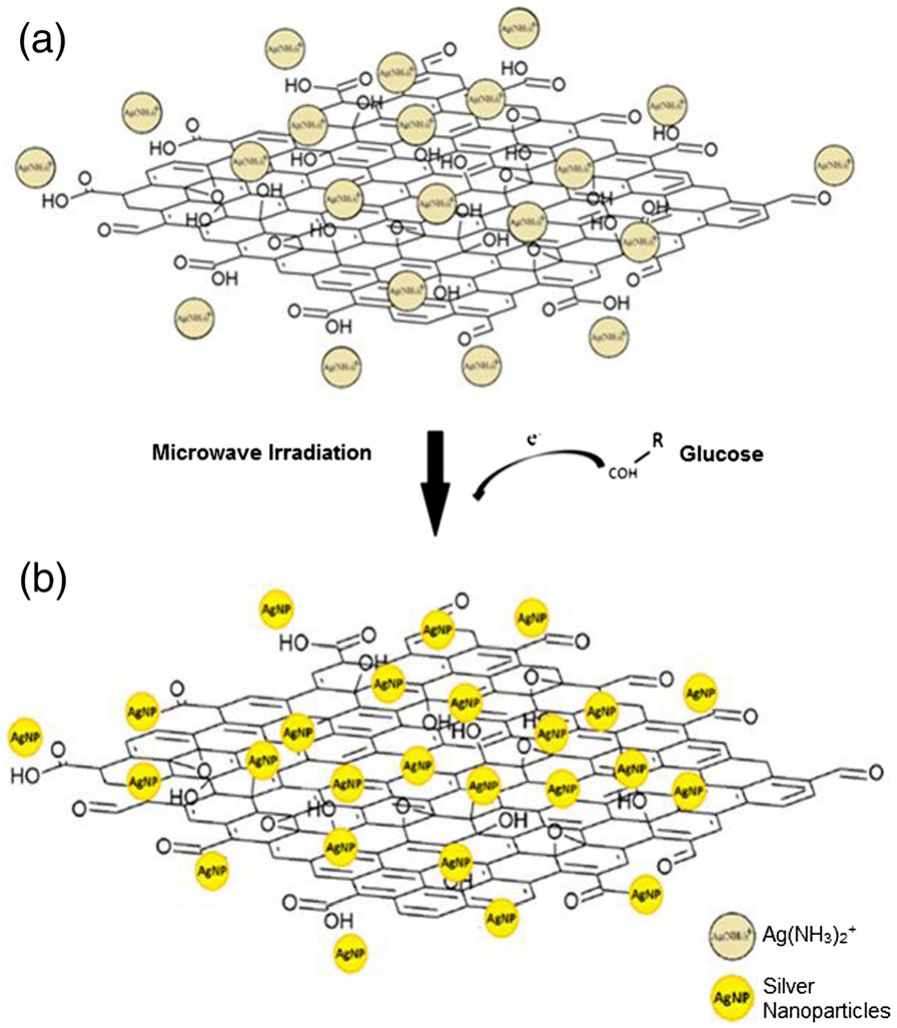
**The formation mechanism of AgGO nanocomposite using microwave irradiation. **(**a**) The electrostatic interaction of Ag(NH_3_)_2_^+^ with oxygenated functional group on the GO sheets and (**b**) the addition of glucose to reduce AgNP under microwave irradiation.

The mechanism of the AgGO formation is shown in Figure 4. Before the reduction by glucose, the positively charged silver ammonia complex, Ag(NH_3_)_2_^+^ can be easily attracted to the negatively charged oxygen functional group on GO (Figure 4a) [25]. After the addition of glucose into the mixture, the aldehyde groups of glucose will release electron to reduce silver ammonia complex. AgNP can then be easily deposited on the GO sheets once the complex is reduced due to the electrostatic attraction between the silver ammonia complex and GO sheets (Figure 4b).

### Characterizations of AgGO

**Figure 5:**
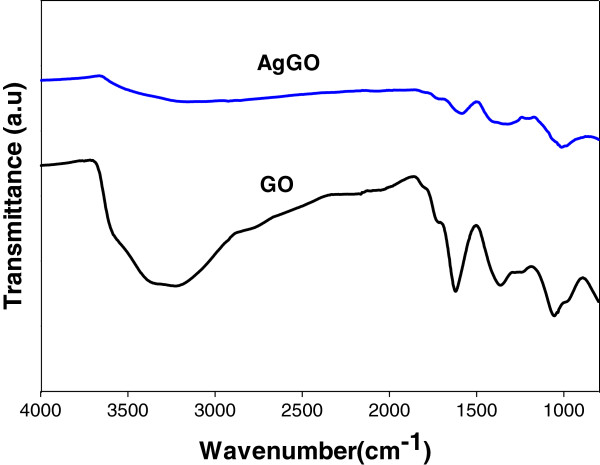
FTIR spectra of GO and AgGO.

The Fourier transform infrared (FTIR) spectra of GO and AgGO are shown in Figure 5. The presence of the adsorption band at approximately 1,619 cm^−1^ corresponds to the C=C bonding of the aromatic rings of the GO carbon skeleton structure. The presence of other oxygenated functional groups can also be detected, including OH at approximately 3,225 and 1,361 cm^−1^, C=O at approximately 1,714 cm^−1^, C-OH at approximately 1,209 cm^−1^, and C-O at approximately 1,054 cm^−1^. Meanwhile, there is a significant decrease in the intensity of the adsorption bands of the oxygenated functional groups for the AgGO sample (Figure 5). This can be due probably to the existence of AgNP on the surface of GO and also to the slight reduction of GO by glucose during the synthesis of the AgGO nanocomposite [37].

**Figure 6:**
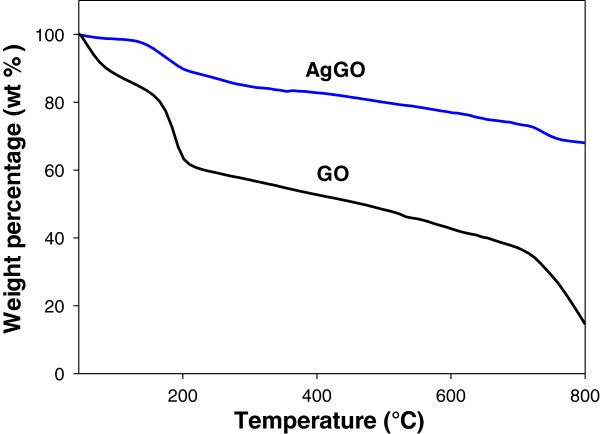
TGA of GO and AgGO.

TGA was conducted for both GO and AgGO samples at temperatures ranging from 30°C to 800°C (Figure 6). The TG curve of the GO sample (Figure 6) shows a major weight loss (approximately 12 wt.%) below 100°C, which can be attributed to the removal of absorbed water [37]. As the temperature increased, the GO has lost approximately 22 wt.% due to the pyrolysis of oxygenated functional groups [38]. On the other hand, AgGO underwent similar weight loss within the same temperature range at a lower rate than that of the GO sample (Figure 6). This can be attributed to the reduction of thermally unstable oxygenated functional groups on the AgGO sample, which is consistent with the FTIR results. The greater thermal stability of the AgGO sample may also be due to the existence of AgNP.

**Figure 7:**
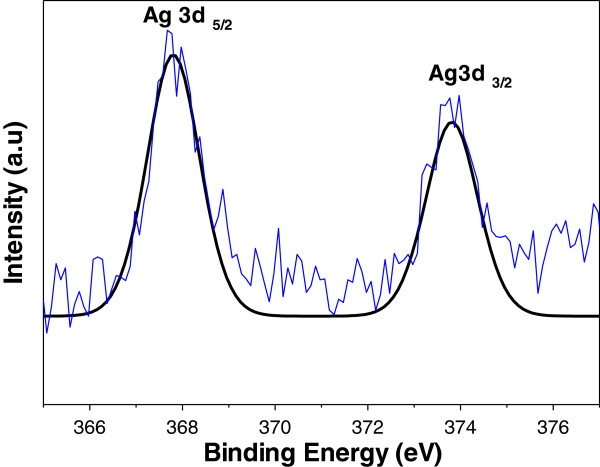
**XPS elemental analysis of Ag 3 *d *in AgGO. **

X-ray photoelectron spectroscopy (XPS) was used to analyze the binding energy of Ag 3*d* in the AgGO sample. The intensity and curve fitting spectra are shown in Figure 7. The existence of two binding energies for Ag 3*d* for the AgGO sample, 367.8 and 373.8 eV, with a difference of 6.0 eV, proved the formation of metallic silver. The shift of both Ag 3*d*_5/2_ and Ag 3*d*_3/2_ (standard binding energies for pure silver are 368.1 and 374.1 eV) to the lower binding energy may be due to the occurrence of electron transfer between AgNP and GO. The results obtained from the TGA and FTIR suggested that the GO was partially reduced during the synthesis of AgGO. Therefore, the disrupted conductivity of GO might be restored during the reduction process by glucose and subsequently enhance the electron movement from Ag within the GO sheets [35].

### Antibacterial activity of AgNP and AgGO

Both AgNP and AgGO nanocomposites were tested for their antibacterial activity against bacteria at five different concentrations, i.e., 100, 50, 25, 12.5, and 6.25 μg/ml. Sterilized distilled water was used as a control sample (Additional file 1). After the incubation, all plates were observed for bacteria colony growth on the surface of the agar medium (Additional files 2, 3, 4, and 5). The observation was recorded and summarized in Table 1. As shown in Table 1, both the AgNP and AgGO samples generally exhibit antibacterial activity that is stronger against Gram-negative than against Gram-positive bacteria. AgGO effectively inhibits *S. typhi* growth at a relatively low concentration of 6.25 μg/ml; nevertheless, 69 colonies were still viable at 6.25 μg/ml in the case of AgNP. For *E. coli*, all tested concentrations of AgNP were inhibitory; however, the lower concentrations of 12.5 and 6.25 μg/ml yielded some surviving colonies, with less than five colonies counted. The results suggest that both AgNP and AgGO are effective anti-Gram-negative bacteria nanomaterials.

**Table 1:** Inhibitory effect of AgNP and AgGO against Gram-positive and Gram-negative bacteria

**Bacteria**	**Sample concentrations (μg/ml)**											**Control**
	**AgNP**					**AgGO**					**GO**	
	**100**	**50**	**25**	**12.5**	**6.25**	**100**	**50**	**25**	**12.5**	**6.25**	**100**	-
Gram-positive												
*S. aureus*	×	×	×	×	×	×	×	×	×	×	×	×
*S. epidermidis*	√	√	×	×	×	×	×	×	×	×	×	×
Gram-negative												
*E. coli*	√	√	√	√	√	√	√	√	√*	√*	×	×
*S. typhi*	√	√	√	√	×	√	√	√	√	√	×	×

A check indicates positive inhibition effect with no colony growth observed. A check with an asterisk indicates positive inhibition effect with less than five colonies observed. An ex indicates negative inhibition effect.

On the contrary, both AgNP and AgGO samples show contrasting effects on the Gram-positive bacteria *S. aureus* and *S. epidermidis*. Both AgNP and AgGO were not effective against *S. aureus* even at the highest tested concentration of 100 μg/ml. The growth of *S. epidermidis* was inhibited by AgNP at 100 and 50 μg/ml, but not at lower concentrations. However, AgGO failed to inhibit the bacteria even at the highest concentration tested.

The interesting result from Table 1 shows that neither Gram-positive nor Gram-negative bacteria exhibit any sign of decrease or inhibitory growth after incubation with a relatively high concentration of GO (100 μg/ml) (Additional file 1), unlike in previously reported works [17-19].

Antibacterial properties of Ag have been widely reported [7,39,40]. AgNPs are attached to the surface of bacteria's membrane and disturb its permeability and respiration ability during their interaction [7]. Furthermore, AgNP has a higher tendency to react with phosphorus and sulfur compounds in bacteria such as the membrane and DNA, causing the bacteria to lose its ability to replicate [39]. Additionally, Ag ions released from AgNP could penetrate into bacteria and cause damage on bacterial main components such as the peptidoglycan, DNA, and protein. These would then lead to the malfunction of the bacterial replication system [41].

The Ag content of AgGO obtained by ICP-OES is 39.5%, which is close to the EDS result (42.5%). This means that a comparable antibacterial performance could be achieved using AgGO with a lower concentration of Ag, as compared to the lone AgNP. AgGO is postulated to have a similar mechanism with that of AgNP with the additional effect from GO. The synergistic effect of AgGO may begin with the wrapping mechanism by the flexible GO sheets, which provides a large surface area substrate for the deposition of AgNP. According to previous studies, GO could adhere to or wrap on the *E. coli* through hydrogen bonds between the bacteria's lipopolysaccharides and the oxygenated functional groups of GO [21,23]. Hence, GO could prevent the nutrient-uptaking process of the bacteria from the surrounding while increasing the interaction between AgNP and the bacteria [21]. The close interaction inflicts a chain reaction starting with the damage and disruption of the bacterial membrane by AgNP, followed by the chemical interaction between the AgNP and the sulfur- and phosphorous-containing compounds of the bacteria [7,39]. Besides, Ag ions can be released more easily into the bacteria, causing mutilation on the respiration and replication of bacteria, and eventually leading to bacterial cell death [42].

**Figure 8:**
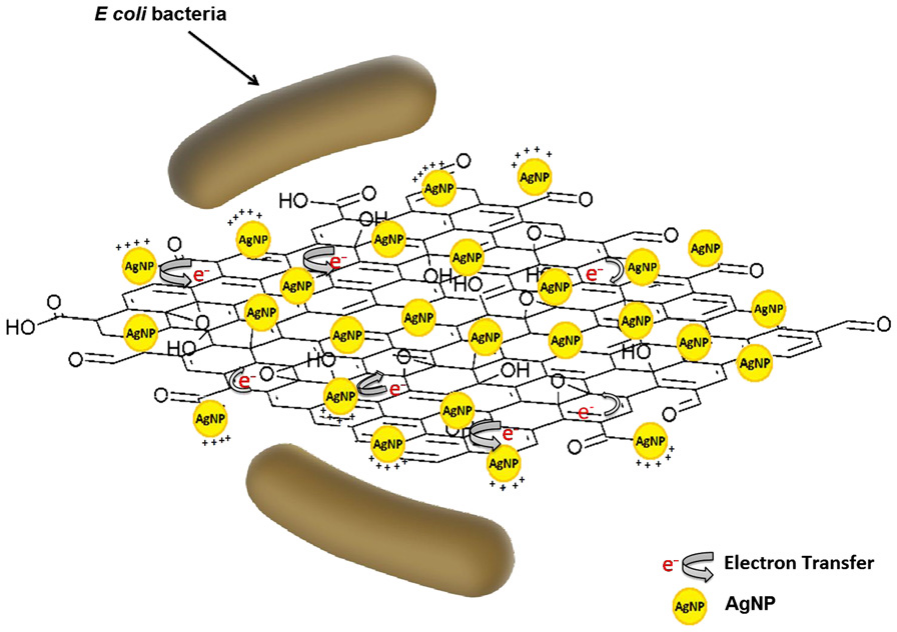
**Plausible antibacterial mechanism of AgGO against *E. coli. ***

The ATR-FTIR results suggest that the GO sheets of the AgGO sample were partially reduced by glucose during the formation and deposition of AgNP on GO. This reduction caused the restoration of the electroconductivity of GO due to the removal of oxygenated functional groups. Moreover, the XPS result of the AgGO [35] indicates the shifting of Ag 3*d* towards the lower binding energy due to the interaction and electron transfer between the metallic Ag and carboxyl groups of the GO sheets. GO with restored electroconductivity could increase the electron transfer rate from the AgNP to the GO, leading to the formation of partially positively charged AgNP, which can enhance the inhibition effect on the cell growth of bacteria. The plausible mechanism above is presented in Figure 8.

The difference in the antibacterial activities of both samples may be due to the structural difference between Gram-positive and Gram-negative bacteria, whereby the former bacteria have a thicker peptidoglycan layer at the outer cell [2]. This is also in agreement with a previous study which reported that AgNP possesses antibacterial effect that is stronger against *E. coli* than *S. aureus* due to the thinner peptidoglycan layer of Gram-negative bacteria [40]. Therefore, the thicker peptidoglycan layer of Gram-positive bacteria might provide protection against the attack by AgNP.

## Conclusions

Microwave irradiation provides a rapid and green method for the synthesis of AgNP and AgGO. It favors the formation of small and uniform nanoparticles through a fast and homogeneous nucleation and crystallization. Both AgNP and AgGO nanocomposites showed antibacterial activity that is stronger against Gram-negative bacteria (*E. coli* and *S. typhi*) than against Gram-positive bacteria, (*S. aureus* and *S. epidermidis*). Meanwhile, the results showed that GO did not exhibit antibacterial activity. The synergistic effect between GO and AgNP has reduced the Ag content without compromising the antibacterial performance. The advantage of this nanocomposite with low Ag content will reduce the concern and risk of excessive Ag usage, which make it a potential material for food packaging and wound dressing applications.

## Competing interests

The authors declare that they have no competing interests.

## Authors’ contributions

SWC, CHC, HMN, and KLC conceived and designed all the experiments. SWC performed all the experiments. RMFRAR helped SWC in performing the antibacterial experiments. RJ and HMN helped SWC in interpreting the antibacterial tests of the samples. SZ, MKA, NMH, and HNL participated in the discussion. All authors read and approved the final manuscript.

## Supplementary Material

Additional file 1 **Table S1.** Digital images for antibacterial effect of water control and GO. Click here for file

Additional file 2 **Table S2.** Digital images for antibacterial effect of AgNP and AgGO against *Staphyloccocus aureus*. Click here for file

Additional file 3 **Table S3.** Digital images for antibacterial effect of AgNP and AgGO against *Staphyloccocus epidermidis. * Click here for file

Additional file 4 **Table S4.** Digital images for antibacterial effect of AgNP and AgGO against *Escherichia coli. * Click here for file

Additional file 5 **Table S4.** Digital images for antibacterial effect of AgNP and AgGO against *Salmonella typhi.* Click here for file
