# Supplementary material for: Antibacterial performance of Ag nanoparticles and AgGO nanocomposites prepared via rapid microwave-assisted synthesis method
Source: Nanoscale Res Lett. 2012 Sep 28;7(1):541. doi: 10.1186/1556-276X-7-541 (PMC3492123; doi:10.1186/1556-276X-7-541)
Supplement: Additional file 1 — Table S1. Digital images for antibacterial effect of water control and GO. [file 1556-276X-7-541-S1.docx]

**Table 1 Digital images for antibacterial effect of water control and GO**

| **Sample** | ***Staphyloccocus aureus*** | ***Staphyloccocus epidermidis*** | ***Escherichia coli*** | ***Salmonella typhi*** |
| --- | --- | --- | --- | --- |
| **Water Control** | 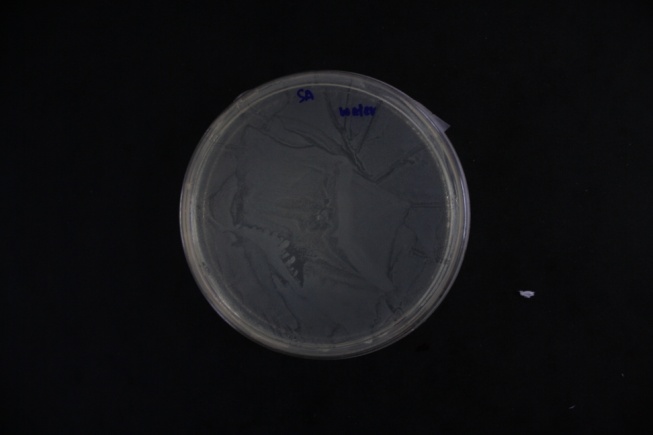 | 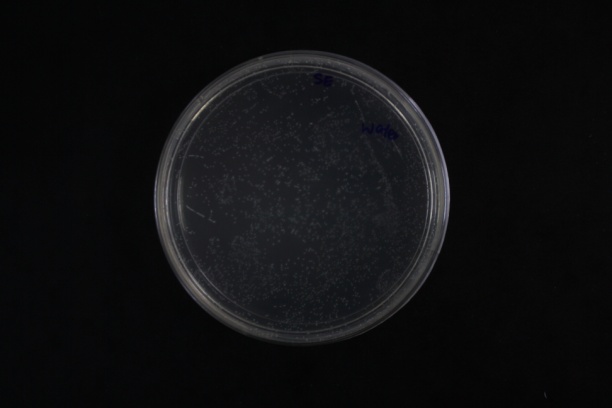 | 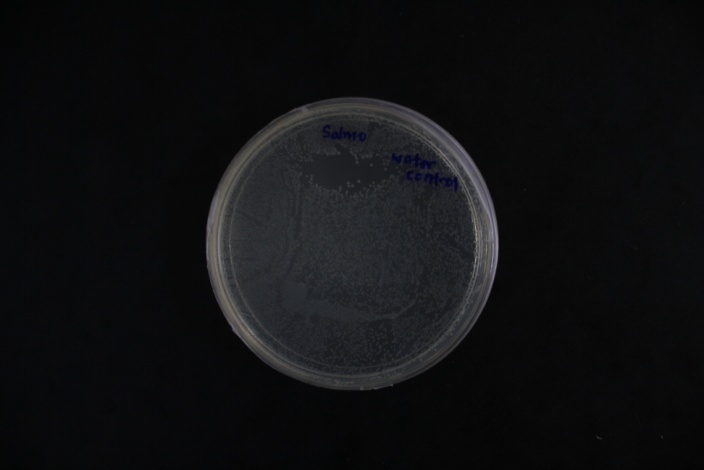 | 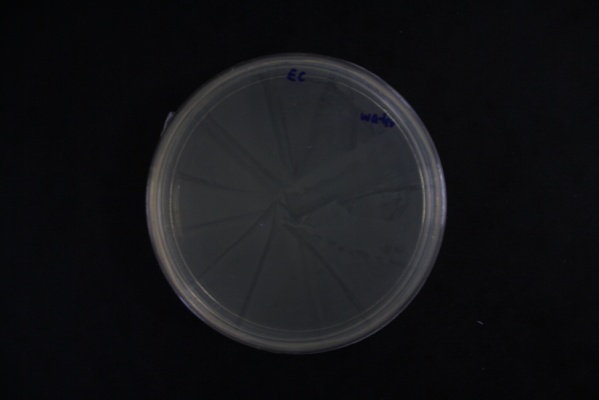 |
| **GO**  **(100 µg/ml)** | 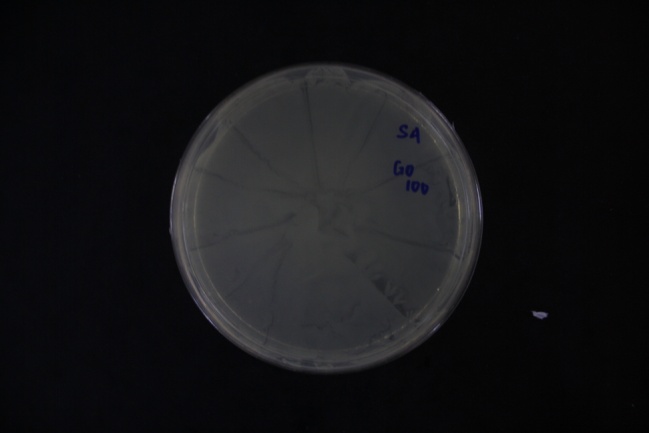 | 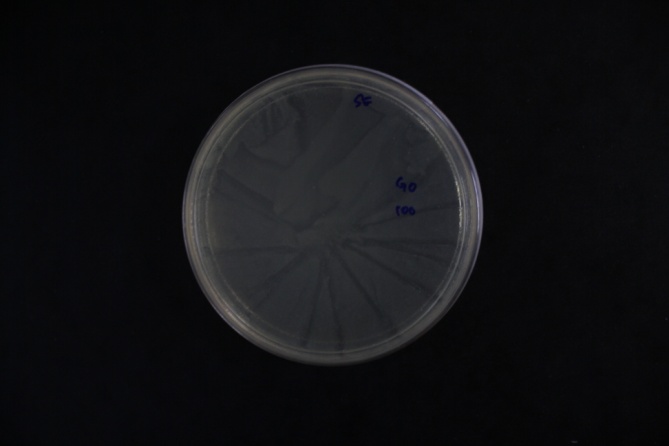 | 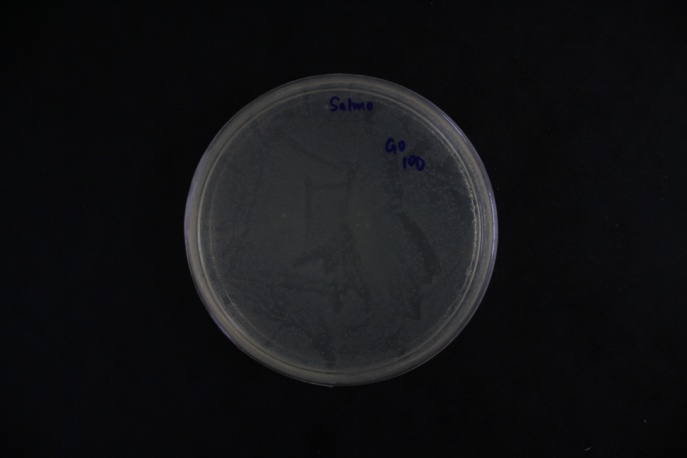 | 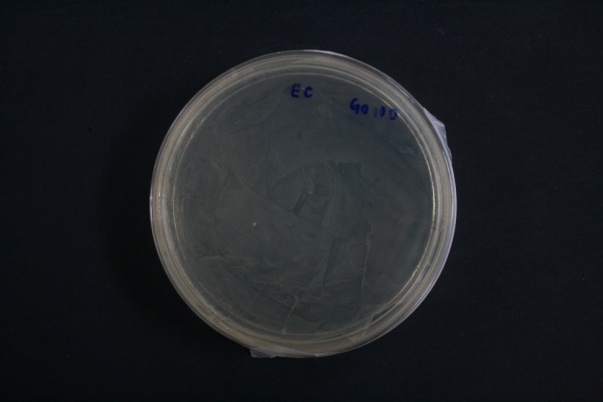 |
