# Supplementary material for: Antibacterial performance of Ag nanoparticles and AgGO nanocomposites prepared via rapid microwave-assisted synthesis method
Source: Nanoscale Res Lett. 2012 Sep 28;7(1):541. doi: 10.1186/1556-276X-7-541 (PMC3492123; doi:10.1186/1556-276X-7-541)
Supplement: Additional file 3 — Table S3. Digital images for antibacterial effect of AgNP and AgGO against Staphyloccocus epidermidis. [file 1556-276X-7-541-S3.docx]

**Table 3 Digital images for antibacterial effect of AgNP and AgGO against *Staphyloccocus epidermidis***

| **Concentrations** | **100 µg/ml** | **50 µg/ml** | **25 µg/ml** | **12.5 µg/ml** | **6.25 µg/ml** |
| --- | --- | --- | --- | --- | --- |
| **AgNP** | 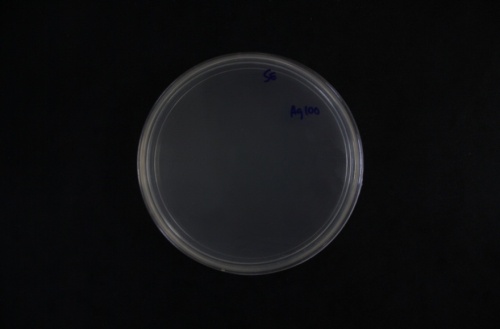 | 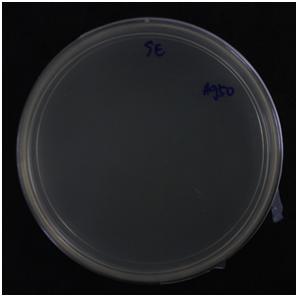 | 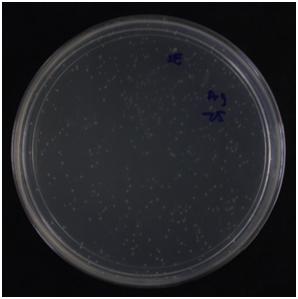 | 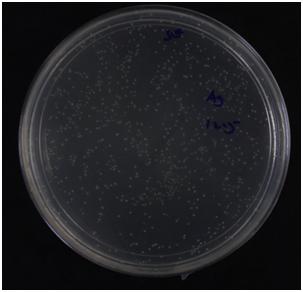 | 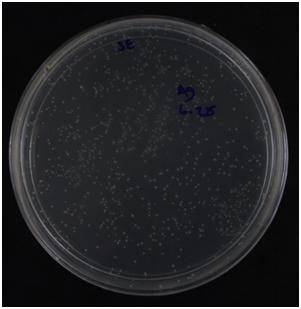 |
| **AgGO** | 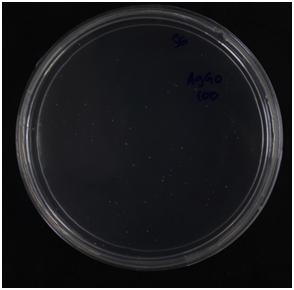 | 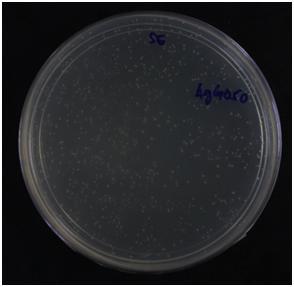 | 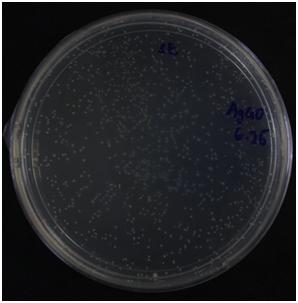 | 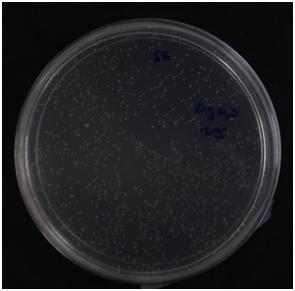 | 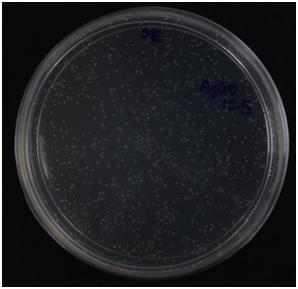 |
